# Aqueous Fraction of *Nephelium ramboutan-ake* Rind Induces Mitochondrial-Mediated Apoptosis in HT-29 Human Colorectal Adenocarcinoma Cells

**DOI:** 10.3390/molecules17066633

**Published:** 2012-05-31

**Authors:** Chim Kei Chan, Bey Hing Goh, Muhamad Noor Alfarizal Kamarudin, Habsah Abdul Kadir

**Affiliations:** Biomolecular Research Group, Biochemistry Program, Institute of Biological Sciences, Faculty of Science, University of Malaya, 50603 Kuala Lumpur, Malaysia

**Keywords:** Nephelium* ramboutan-ake*, pulasan, cytotoxicity, apoptosis

## Abstract

The aim of this study was to investigate the cytotoxic and apoptotic effects of *Nephelium ramboutan-ake* (pulasan) rind in selected human cancer cell lines. The crude ethanol extract and fractions (ethyl acetate and aqueous) of *N. ramboutan-ake* inhibited the growth of HT-29, HCT-116, MDA-MB-231, Ca Ski cells according to MTT assays. The *N. ramboutan-ake* aqueous fraction (NRAF) was found to exert the greatest cytotoxic effect against HT-29 in a dose-dependent manner. Evidence of apoptotic cell death was revealed by features such as chromatin condensation, nuclear fragmentation and apoptotic body formation. The result from a TUNEL assay strongly suggested that NRAF brings about DNA fragmentation in HT-29 cells. Phosphatidylserine (PS) externalization on the outer leaflet of plasma membranes was detected with annexin V-FITC/PI binding, confirming the early stage of apoptosis. The mitochondrial permeability transition is an important step in the induction of cellular apoptosis, and the results clearly suggested that NRAF led to collapse of mitochondrial transmembrane potential in HT-29 cells. This attenuation of mitochondrial membrane potential (Δ*ψ*m) was accompanied by increased production of ROS and depletion of GSH, an increase of Bax protein expression, and induced-activation of caspase-3/7 and caspase-9. These combined results suggest that NRAF induces mitochondrial-mediated apoptosis.

## 1. Introduction

Cancer is a major health problem of global concern and is the second leading cause of death. Among various types of cancer, colorectal cancers are the third most common cancer in both men and women [1]. Multiple steps are required for the process of cancer development which enables normal cells to turn into tumors through the initiation phase, followed by the promotion stage leading to malignant growth and invasion in the progression stage [2]. For colorectal carcinomas, normal colonic epithelium transforms into carcinoma through adenoma as intermediate. This progression is known as the adenoma-carcinoma sequence. Colorectal tumorigenesis arises as a consequence of the progression of histological changes and concurrent accumulation of genetic and epigenetic alterations. The most prominent genes involved in these alterations include APC, K-ras, p53, c-myc, DNA mismatch repair genes, BRAF, PIK3CA and PTEN. Accumulation of these alterations promotes the growth and results in clonal expansion of tumor cells [3,4]. 

Apoptosis or programmed cell death plays a crucial role in tissue development and homeostasis which is characterized by a series of morphological and biochemical changes such as nuclear condensation, DNA fragmentation, membrane blebbing, phosphatidylserine externalization and loss of mitochondrial membrane potential [5,6]. Additionally, mitochondria are found to play a crucial role in the regulation of apoptosis. A loss in mitochondrial membrane potential results in the translocation of proapoptotic Bax to mitochondria and the release of cytochrome c into cytosol which subsequently lead to the activation of caspase cascades [7]. Numerous studies had reported that oxidative stress as a result of excessive production of reactive oxygen species (ROS) and depletion of glutathione level contribute in the initiation of apoptotic signaling [8,9].

The balance between cell proliferation and apoptosis in colonic mucosa is tightly regulated in order to maintain a constant cell number. The perturbation in the balance between cell proliferation and apoptosis in colonic mucosa causes an escape from the normal homeostasis of cell number along with the progression of cancer cells [10,11]. Hence, inhibition of proliferation and increase in apoptosis of these aberrant cells are the key mechanisms in preventing colon cancer. Above and beyond, apoptosis also presents as a window that can be exploited for the development of potential therapeutic drugs. Currently, in the eradication of cancers, the most common clinical therapeutic treatments currently available to improve patient prognosis include chemotherapy, radiotherapy and surgery which are associated with toxicity and the occurrence of resistance constraint their effectiveness. Moreover, after surgical resection, some of the patients with colorectal cancer require adjuvant therapy. Therefore, there is an urgent demand and interest to develop novel treatment by using plant-derived novel anticancer agents which are effective and with minimal side-effects [12].

Recently, it has been found that the intake of fresh fruits, vegetables and plants rich in natural antioxidants could be associated with the prevention of diseases such as cancer, diabetes mellitus and cardiovascular diseases [13,14]. Tropical fruits such as mangosteen, rambutan, mango, pomegranate and others are lately found to contain large variety of substances possess antioxidant [15,16] anti-diabetic [17] and anti-cancer properties [16,18]. Interesting recent research has revealed that rinds of various fruits such as grapes, pomegranate, mangosteen and rambutan possess antioxidant properties [15]. Thus, the rind *N. ramboutan-ake *(known commonly in Peninsular Malaysia as pulasan), which has shown strong antioxidant activities in our previous study (data not shown) has become the target of interest in our current research. *N. ramboutan-ake *is a tropical fruit, in which the rind is normally discarded as waste after consumption. *N. ramboutan-ake *is a plant from the Sapindaceae family which is closely allied to the rambutan. The root decoction of *N. ramboutan-ake *is traditionally used for treating feverish patients. According to present knowledge, studies on isolation and identification of compounds in closely allied plants of the same genus, namely *N. lappaceum* and *N. maingayi* have revealed the presence of bioactive compounds of different chemical types, including tannins (geraniin and corilagin), phenolic acid (ellagic acid) [19] and saponins (7*R*-methoxyerythrodiol, erythrodiol, maniladiol) [20]. Geraniin, a hydrolysable ellagitannin, is found to possess anticancer activities against human melanoma cells [21], erythrodiol was implied in anticancer activities against astrocytomas [22] while ellagic acid was involved in anticancer activities against human bladder cancer T24 cells and human colon adenocarcinoma Caco-2 cells [23,24]. In our previous study, NRAF has been found to possess potent antioxidant activity (unpublished data), which led us to investigate its cytotoxic properties. To the best of our knowledge, the effect of *N. ramboutan-ake *on human cancer cell lines has not been reported. Thus, in the present study, we have investigated the cytotoxic effects of NRAF in HT-29 human colon adenocarcinoma cancer cells. In addition, we also examined the effects of NRAF on apoptosis and suggest a possible NRAF-induced apoptotic mechanism for the observed activity. 

## 2. Results and Discussion

### 2.1. Reduction of HT-29 Cell Viability by NRAF

**Figure 1:**
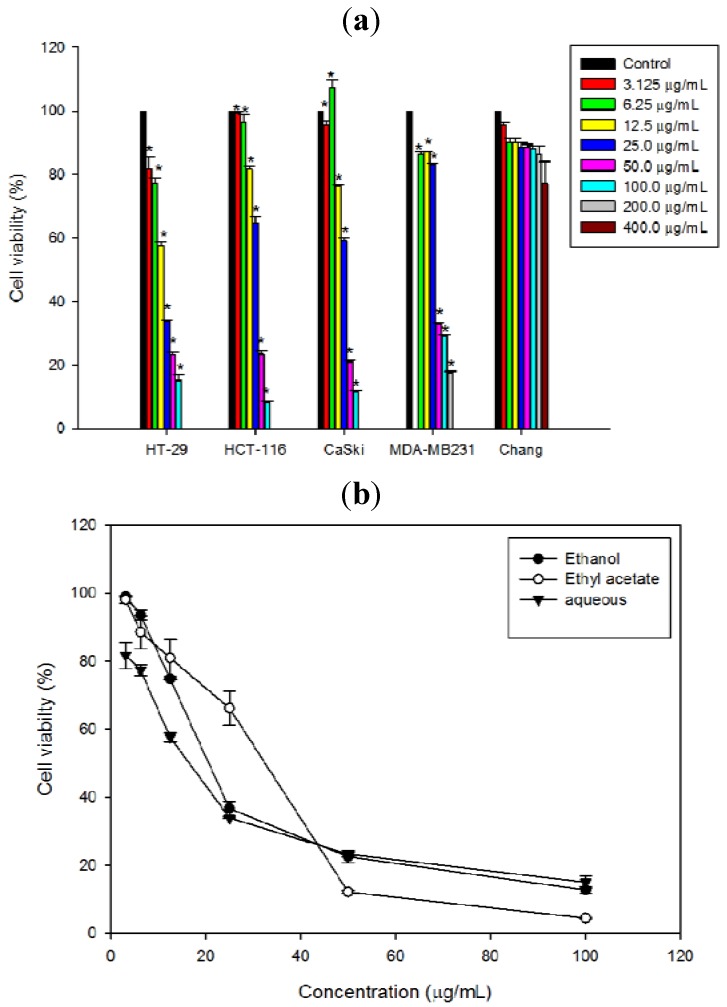
The cytotoxicity of NRAF extract and fractions against selected cancer cell lines at 72 h. (**a**) represented the effect of NRAF on the viability of HT-29, HCT-116, Ca Ski, MDA-MB-231 and Chang liver cells. (**b**) Represented the cytotoxicity of *N. ramboutan-ake* rind ethanol extract (NREE), ethyl acetate fraction (NREAF) and aqueous fraction (NRAF) against HT-29 cell lines. The data expressed as mean ± S.E. of three independent experiments (n = 9). Asterisks indicate significantly different value from control (* *p* < 0.05).

The cytotoxicity of *N. ramboutan-ake* rind on various cancer cells was evaluated based on the cell viability determined using MTT assays. The results indicated that the crude ethanol extract and fractions of *N. ramboutan-ake* rind reduced the cell viability of four different cell lines namely, Ca Ski cells, MDA-MB-231, HT-29 cells and HCT-116 cells in a dose-dependent manner (Figure 1a). Among the fractions, NRAF displayed the greatest growth inhibitory effect against HT-29 cells in a dose-dependent manner, followed by NREE and NREAF (Figure 1b). The IC_50_ values were 16.66 ± 0.55 µg/mL, 20.70 ± 0.49 µg/mL, 32.24 ± 1.81 µg/mL for NRAF, NREE and NREAF, respectively (Table 1). NRAF yielded a lower IC_50_ value than curcumin, used as a positive control in this study (Table 1). Clearly, HT-29 was the most susceptible cell line and thus it was selected for further investigations. NRAF also exhibited a significant dose-dependent reduction of cell viability on Ca Ski (31.14 ± 0.41 µg/mL), HCT-116 (33.90 ± 1.06 µg/mL) and MDA-MB231 (41.53 ± 0.32 µg/mL) while for Chang liver cells, as representative of normal cells, a decrease in cell viability was only observed at 400 µg/mL.

### 2.2. Preliminary Phytochemical Screening

In the present study, a qualitative phytochemical analysis of NRAF revealed the presence of flavonoids, tannins and saponins, while showing no presence of alkaloids and sterols (Table 2). This was observed through the colour intensity, turbidity and precipitation seen in the different reactions. Meanwhile, the results of the present study indicated that NRAF exhibited the greatest cytotoxicity against HT-29, which may be associated with the high levels of tannins and saponins in this fraction. In this context, further study is required to identify the molecules responsible for this bioactivity. Comprehensive phytochemical analysis for the isolation and identification of active compounds in NRAF is currently undertaken to provide a rational conclusion on its usage as a potential anticancertherapeutic agent.

**Table 1:** IC_50_ values of extract and fractions of *Nephelium ramboutan-ake *rind against different cancer cell lines.

Cell lines	IC_50_ (µg/mL)				
	Ethanol	Ethyl acetate		Aqueous	Curcumin *
HT-29	20.70 ± 0.49	32.24 ± 1.81	16.67 ± 0.55		21.32 ± 0.17
HCT-116	35.73 ± 0.56	47.23 ± 2.84	33.90 ± 1.06		NA
Ca Ski	34.38 ± 0.66	44.90 ± 0.58	31.14 ± 0.41		NA
MDA-MB-231	51.09 ± 1.32	61.65 ± 0.42	41.53 ± 0.32		NA

The data represent mean ± S.E. of three independent experiments (n = 9). NA: Not available. * Curcumin served as positive control.

**Table 2:** Preliminary phytochemical analysis of *N. ramboutan-ake* aqueous fraction (NRAF).

Phytochemical test	Results *
Flavonoids	++
Tannins	+++
Saponins	+++
Alkaloids	−
Sterols	−

* − absent; + low, ++ moderate and +++ abundant.

### 2.3. Induction of Nuclear Morphological Changes by NRAF

**Figure 2:**
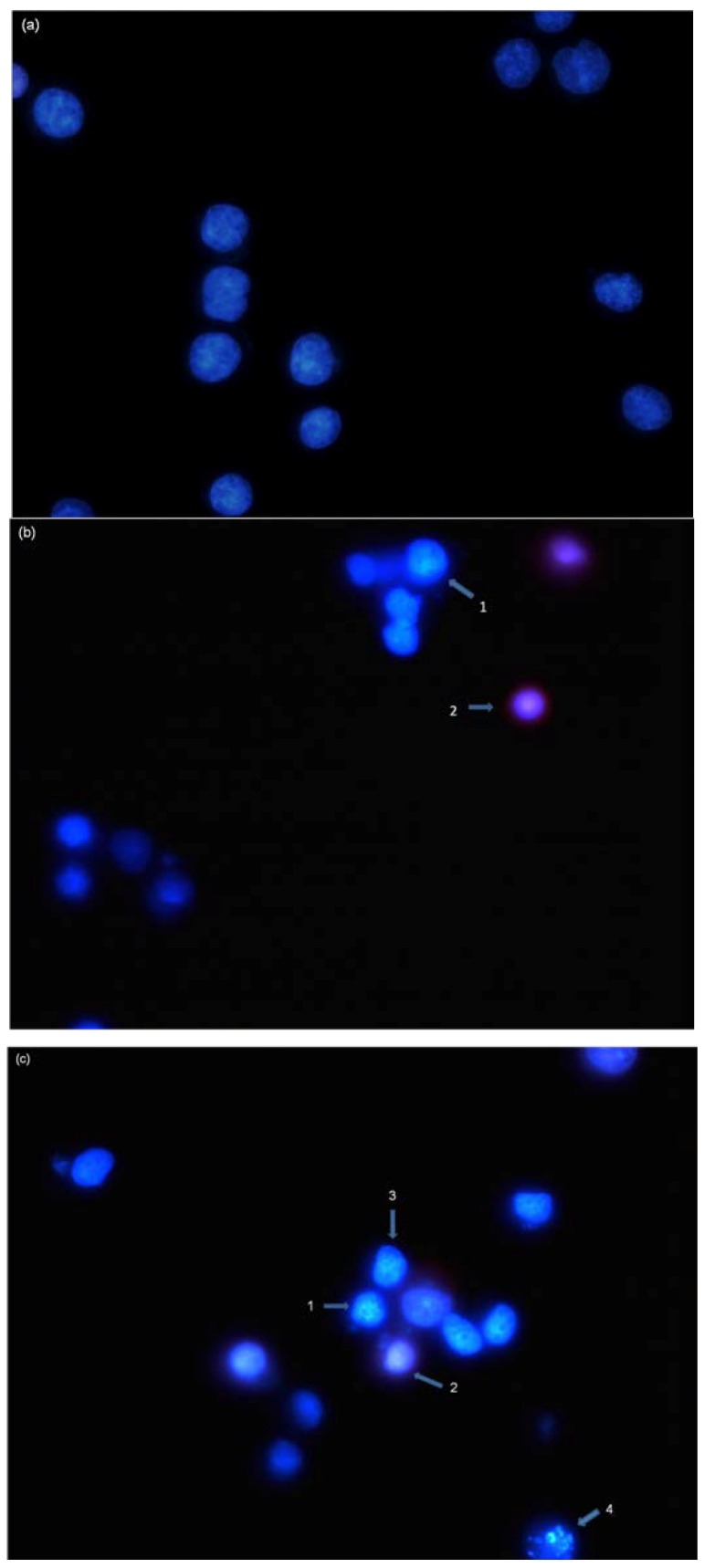
Nuclear morphological changes of HT-29 cells by NRAF. (**a**) Untreated control cells. After exposure to 50 µg/mL of NRAF. (**b**) and 100 µg/mL of NRAF. (**c**) for 24 h, when stained with Hoechst 33342 and PI. Arrows indicated early (e.g., chromatin condensation, cell shrinkage and nuclear fragmentation) and late apoptotic morphological changes. Magnification: 630×. Arrow 1 indicates chromatin condensation, 2 late apoptosis, 3 cell shrinkage, 4 DNA fragmentation.

To further investigate whether NRAF-mediated cell death in HT-29 cells was due to apoptosis, the cells were double stained with Hoechst 33342/propidium iodide (PI) and the resulting nuclear morphological changes were observed under a fluorescence microscope. Hoechst is a DNA stain that binds preferentially to T-A base pairs. In the control untreated cells, the nuclei appeared to be round and even in shape with intact chromatin were homogenously stained with faint blue fluorescence (Figure 2a). However, after treatment with NRAF (50 µg/mL) for 24 h cells showed an increase in the intensity of blue fluorescence nuclear shrinkage and chromatin condensation (Figure 2b, Arrow 1) typical of early apoptosis. Cells that were dual-stained were considered to be in their late apoptosis stage (Figure 2b, Arrow 2). With increasing concentration of NRAF (100 µg/mL), the nuclear changes were more apparent, exhibiting more obvious cell shrinkage and loss of nuclear architecture (Figure 2c, Arrow 3). This progressed into fragmented nuclei in the process of forming apoptotic bodies (Figure 2c, Arrow 4). Meanwhile, these aberrant nuclear changes were not seen in the untreated control cells. These observations indicated that apoptosis occurred in HT-29 cells following treatment with NRAF.

### 2.4. Induction of DNA Fragmentation Detected by TUNEL Assay

**Figure 3:**
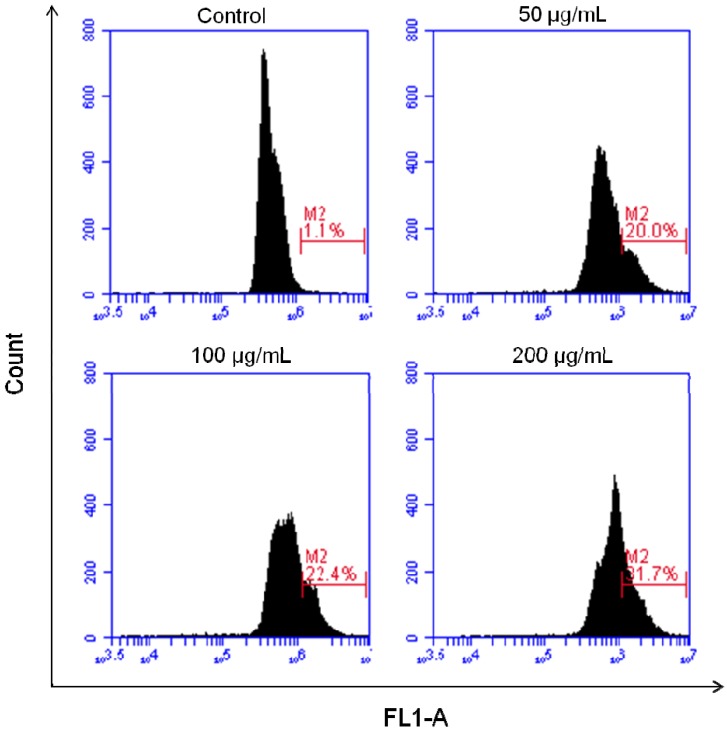
Effect of NRAF on DNA fragmentation of HT-29 cells. HT-29 cells were treated with different concentrations of NRAF (50 µg/mL, 100 µg/mL and 200 µg/mL) at 24 h incubation period. Positive TUNEL staining was shown by the M2 region in which cells were stained with FITC-conjugated anti-BrdU antibody. Histograms are representatives of three separate experiments (n = 3).

According to Kerr and Harmon [25], DNA fragmentation is one of the hallmarks of apoptosis cell death that is induced by most anticancer agents. In the current study, we confirmed the occurrence of DNA fragmentation in HT-29 cells treated with NRAF by a TdT-mediated dUTP Nick End Labelling (TUNEL) assay utilizing APO-BRDU kit (Sigma). The TUNEL assay was developed as a method to identify individual cells that were undergoing apoptosis by labeling the ends of degraded DNA with the polymerase terminal deoxynucleotidyl transferase (TdT) [26], which catalyzes the template-independent addition of deoxynucleotide triphosphates to the 3'-OH ends of DNA. During late stage of apoptosis, endonucleases cause DNA degradation, resulting in fragments of DNA strand breaks (DSBs) with exposed 3'-OH ends [27]. The incorporated BrdUTP was then detected with FITC-conjugated BrdUTP antibody. The control untreated cells showed negative TUNEL staining (Figure 3). However, NRAF-treated cells showed positive TUNEL staining indicating the occurrence of DNA fragmentation. The percentage of TUNEL-positive cells increased with increasing concentrations of NRAF (Figure 3). The cell death percentage (apoptotic index) was markedly increased up to 31.07 ± 0.79% compared to the control group (1.1 ± 0.5%) at 24 h of treatment. These data verify that NRAF induces HT-29 cell apoptosis in a dose-dependent manner. The increase in TUNEL positivity was in parallel with the appearance of apoptotic nuclear morphological alterations as illustrated in Hoechst 33342/PI staining. Thus, we suggest that DNA fragmentation, particularly the breakage of DNA strands, might contribute to the aberrant nuclear changes. Taken together, these observations showed that the cells underwent apoptotic DNA damage after treatment with NRAF.

### 2.5. Induction of Early and Late Apoptosis Using Annexin V/PI Staining.

**Figure 4:**
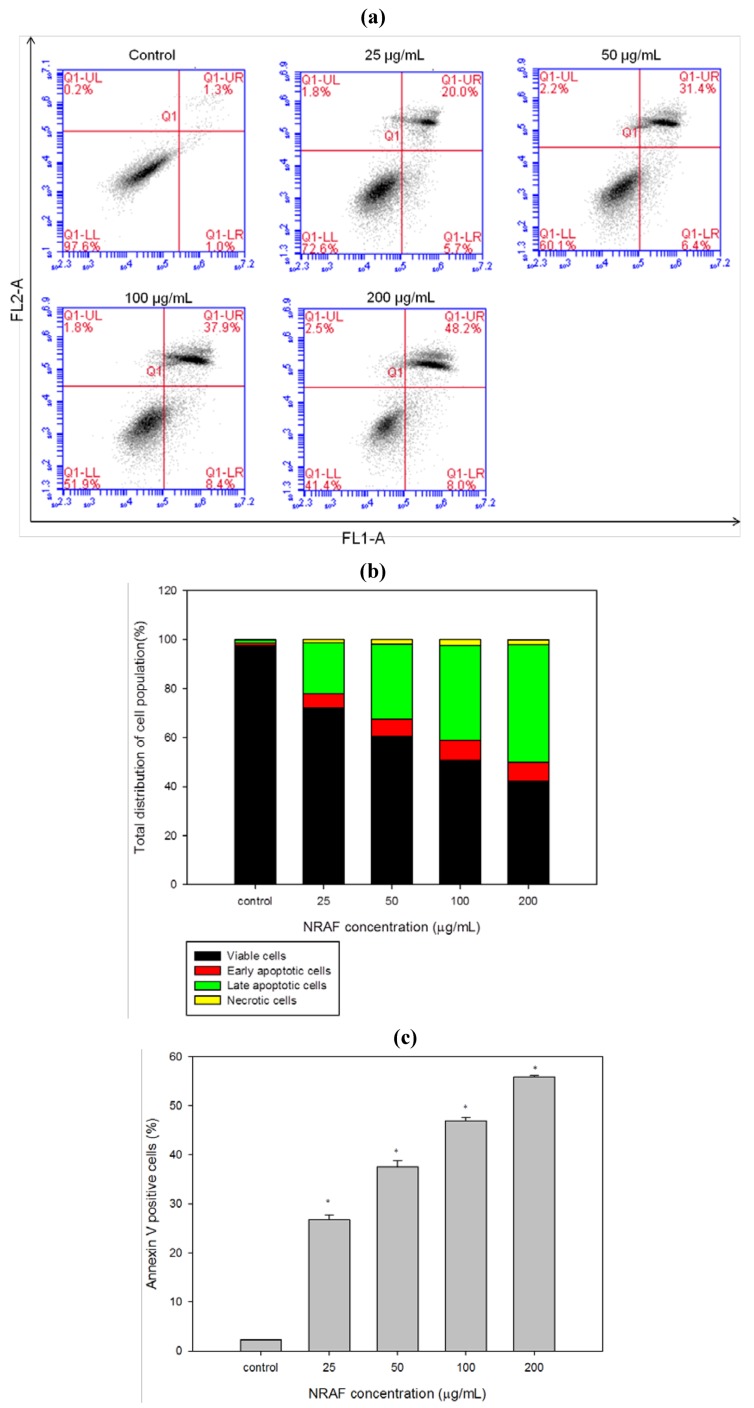
Dose-dependent induction of early and late apoptosis by NRAF (25–200 µg/mL) at 24 h. (**a**) showed the flow cytometric fluorescence patterns of Annexin V-FITC/PI staining. (**b**) indicated the percentage of viable, early apoptotic, late apoptotic and necrotic cells. (**c**) depicted the percentage of annexin V positive cells. The data expressed as mean ± S.E. from three individual experiments. Asterisks indicate significantly different value from control (* *p* < 0.001).

Our results thus far have demonstrated the typical morphological changes that occur during late apoptosis. This led us to investigate the effect of NRAF on early events of apoptosis. Numerous studies have reported that advanced DNA fragmentation is preceded by alterations in the plasma membrane, such as phosphatidylserine (PS) externalization. During early apoptosis, the plasma membrane asymmetry is lost due to the externalization of PS [28], onto the cell surface, which is considered one of the prime markers of apoptosis. Hence, NRAF-treated cells were double stained with Annexin V-FITC/PI and analyzed by flow cytometry. This assay is based on the fact that apoptotic cells have exposed phosphatidylserine molecules [29] and thus bind with annexin Vwhile necrotic cells possess compromised membranes and thus take up PI [30]. Annexin V is a Ca^2+^ dependent phospholipid-binding protein that detects the PS externalization of plasma membrane [31]. The faintly annexin V-stained cells probably due to a limited PS exposure during the early stage of apoptosis [32]. The heavily stained annexin V and PI cells indicated the later stage of the apoptosis, whereby the cells lost their plasma membrane integrity and more binding sites of PS were detected [28]. According to Figure 4a,b, control untreated cells showed low or negative staining with both Annexin V and PI (Annexin V^−^/PI^−^) indicating viable cells. When challenged with increasing concentrations of NRAF for 24 h, the results clearly showed the progression from early to late apoptosis, as indicated by the concentration-dependent accumulation of the late apoptotic cells. Notably, the necrotic cell population has remained low throughout the treatment (below 3%). In this study, we found that the combined early and late apoptotic cells (annexin V positive) increased significantly in a dose-dependent manner compared to the control (Figure 4c), indicating the initiation of apoptosis. Thus, the different stages of apoptosis were observed and we concluded that NRAF caused cell death in HT-29 cells through induction of apoptosis. 

### 2.6. Dissipation of Mitochondrial Membrane Potential (Δψm) Triggered by NRAF in HT-29 Cells

**Figure 5:**
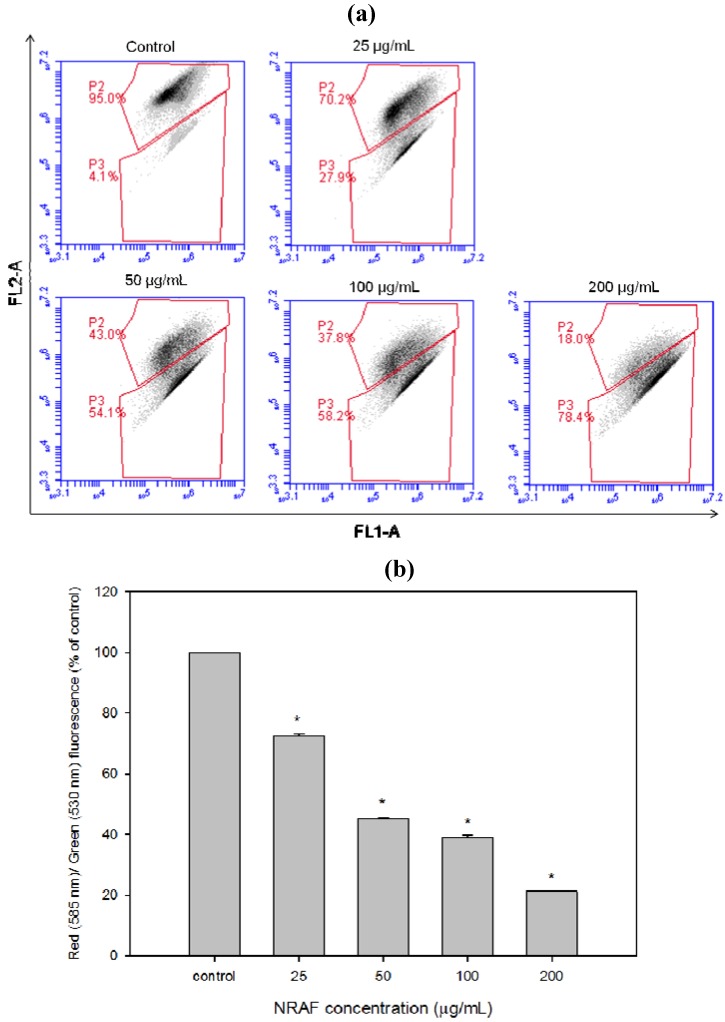
Dose-dependent attenuation of mitochondrial membrane potential in HT-29 cells elicited by NRAF. (**a**) Showed the flow cytometric fluorescence patterns analysis of JC-1 (5,5',6,6'-tetrachloro-1,1',3,3'-tetraethylbenzimidazolylcarbocyanine iodide) staining. (**b**) Showed that NRAF has resulted in a substantial dose-dependent reduction of red/green fluorescence corresponding to loss of *Δψm*. The data expressed as mean ± S.E. from three individual experiments. Asterisks indicate significantly different value from control (* *p* < 0.001).

The disruption of mitochondrial integrity is one of the early events leading to apoptosis. Mitochondrial dysfunction usually triggers specific cellular signaling to induce apoptosis. Increasing evidence suggests that altered mitochondrial function is linked to apoptosis and a decreasing mitochondrial transmembrane potential, *Δψm*, is associated with mitochondrial dysfunction. Therefore, loss of mitochondrial membrane potential is an important event during the mitochondrial-mediated apoptosis [33,34,35], so we investigated whether NRAF could induce loss of mitochondrial membrane potential in HT-29 cells by measuring mitochondrial membrane polarity using the unique fluorescent cationic dye, JC-1 probe. The NRAF-treated cells showed progressive loss of red JC-aggregate fluorescence and appearance of green monomer fluorescence in the cytoplasm at 25 µg/mL and almost complete loss of red fluorescence at 200 µg/mL (Figure 5a). As a result, the treated cells had lower red fluorescence than the untreated cells which corresponded to mitochondria with a loss of *Δψm*. As indicated by JC-1 red/green ratio (Figure 5b), NRAF resulted in a substantial dose-dependent loss of *Δψm*. These data suggest that NRAF-induced apoptosis in HT-29 cells involved mitochondrial dysfunction associated with dissipation of the *Δψm*. 

### 2.7. NRAF Effect on Intracellular ROS and GSH Levels in HT-29 Cells

The changes in *Δψm* can result from oxidative stress-induced apoptotic signaling that is consequent to ROS increases and/or antioxidant decreases, disruption of intracellular redox homeostasis and irreversible oxidative modifications of lipids, proteins, or DNA [36]. Redox homeostasis in a cell is due to a fine balance between the intracellular ROS and ROS scavenging antioxidants and enzyme systems. GSH is an intracellular antioxidant and is known to participate in maintaining cellular redox balance [37]. Production of intracellular ROS has been demonstrated to be an early signal that mediates apoptosis [38], in parallel with *Δψm* loss as an early event in apoptosis induction [28]. 

**Figure 6:**
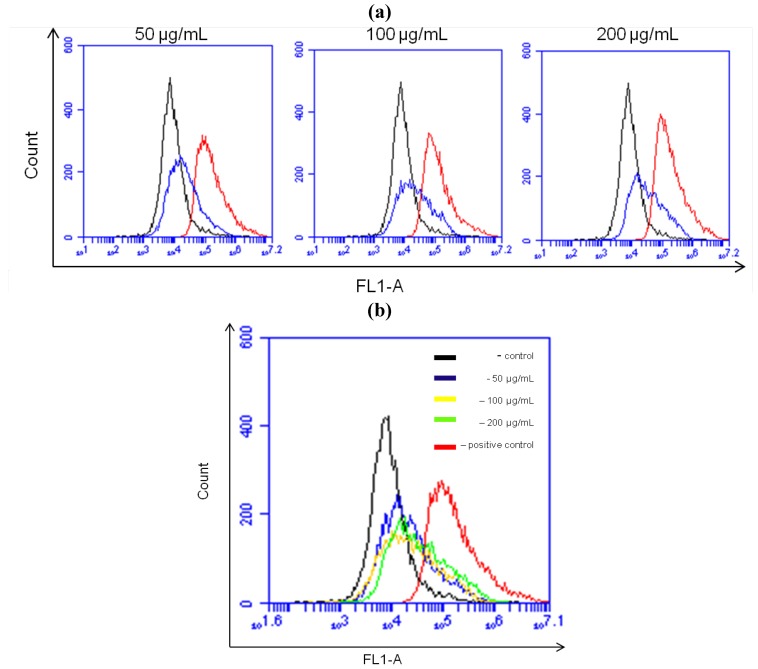
Effect of NRAF on intracellular ROS level. (**a**) Showed formation of ROS was induced after treatment with different concentrations of NRAF (50 µg/mL, 100 µg/mL and 200 µg/mL). (**b**) Data analysis indicating shifts in the intracellular ROS level at different concentrations when compared to the negative control (untreated cells) and positive control (treated with TBHP).

With this in mind, we have proceeded to determine the intracellular levels of ROS and GSH. Since a loss of mitochondrial membrane potential is associated with the generation of ROS [39], we detected the level of intracellular ROS in HT-29 cells treated with various concentrations of NRAF. The level of ROS within the cells was measured using a ROS-sensitive fluorometic probe, 2'-7'-dichloro-fluorescein diacetate (DCFH-DA) indicated by dichlorofluorescein (DCF) fluorescence which was proportional to the amount of intracellular ROS formed. Following treatment with 50 µg/mL of NRAF, a significant generation of ROS appeared as early as 4 h after treatment (Figure 6) as evident by the shift of the histogram from left to right, indicating an increase in the fluorescence intensity (Figure 6a). A progressive shift of the histogram to the right with increasing concentrations of NRAF was also observed as shown in Figure 6b.

**Figure 7:**
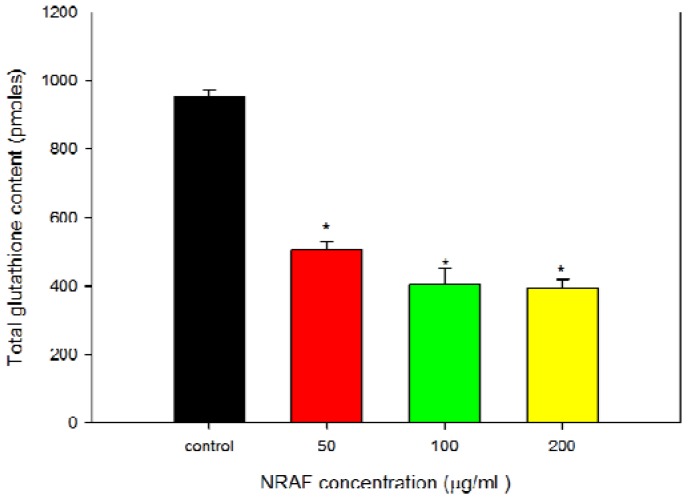
Effect of NRAF on intracellular total glutathione content of HT-29 cells at 24 h. There was a significant reduction in intracellular GSH content (>50%) after treatment with varying concentrations of NRAF (50–200 µg/mL). The data expressed as mean ± SE of three independent experiments (n = 9). Asterisks indicate significantly different value from control (* *p* < 0.001).

In contrast to ROS, intracellular GSH level was seen to decrease with increasing concentrations of NRAF (Figure 7). In order to correlate between the intracellular GSH content and apoptosis, the content of intracellular GSH in NRAF-treated and untreated cells was investigated. The results showed that the intracellular GSH level was high (952.50 ± 21.65 pmoles) in the control untreated cells and has significantly depleted to 505.00 ± 23.63, 404.17 ± 46.93 and 394.17 ± 24.85 pmoles with increasing concentrations of NRAF of 50, 100 and 200 µg/mL, respectively, after 24 h treatment. This result indicated a shift of redox equilibrium towards prooxidant state (Figure 6 and Figure 7). The present findings provided interesting insight into the systematic effect of NRAF on intracellular redox state and apoptosis. This study revealed that the apoptotic mechanism of NRAF in HT-29 cells was partially related to ROS production, GSH depletion and *Δψm* loss. These phenomena are similar to the effects observed for some clinical and pre-clinical anticancer drugs. For example, cisplastin can change the intracellular ROS and *Δψm* in human HepG2 hepatoma cells [40]. Curcumin, a pre-clinical anticancer drug, can increase ROS, induce GSH depletion, decrease *Δψm*, release cytochrome c and activate caspases to induce apoptosis in cancer cells [41]. This study has implied that oxidative stress due to increased intracellular ROS production and GSH depletion subsequently resulted in cellular damage and apoptosis.

### 2.8. Modulation of Apoptotic Proteins by NRAF

Loss of *Δψm* corresponds to mitochondrial dysfunction which in turn determines the mitochondrial permeability transition which is an important step in the induction of cellular apoptosis. Our present findings demonstrated NRAF-induced collapse of the mitochondrial transmembrane potential in colon cancer cells HT-29 (Figure 5) and this collapse is thought to occur through formation of pores in the mitochondria by dimerized Bax or activated Bid, Bak or Bad proteins. Activation of these proapoptotic proteins is accompanied by release of cytochrome c into the cytoplasmwhich promotes the activation of caspases that are directly responsible for apoptosis [42]. In view of that, we investigated mitochondrial-mediated apoptotic mechanism through the expression of Bax and Bcl-2 proteins.

Members of the Bcl-2 family, including proapoptotic (Bid, Bax, and Bak) and antiapoptotic (Bcl-2 and Bcl-xL) proteins are critical regulators of the intrinsic pathway to modulate the permeabilization of mitochondrial membrane [43]. In many human cancers, the antiapoptotic Bcl-2 proteins are overexpressed, or the proapoptotic proteins, like Bax, have reduced expression [44]. This results in resistance to a wide variety of cell death stimuli, including chemotherapeutic drugs [45]. Bcl-2 maintains mitochondrial integrity, while Bax destroys the mitochondrial integrity and causes loss of *Δψm* [46]. Bax exerts proapoptotic activity by translocating from the cytosol to the mitochondria, where it induces cytochrome c release, whereas Bcl-2 exerts its anti-apoptotic activity, at least in part, by inhibiting the translocation of Bax to the mitochondria [47,48]. Other studies have implicated that the elevation of Bax protein is associated with the mitochondrial (intrinsic) pathway in apoptosis which involves the reduction of membrane potential, cytochrome c release and subsequently leading to caspase activation [49,50]. The expression of the proapoptotic protein Bax is an early event that sensitizes the cells to undergo apoptosis. Some models suggest that Bax upregulation alone can commit a cell to apoptosis [51]. 

**Figure 8:**
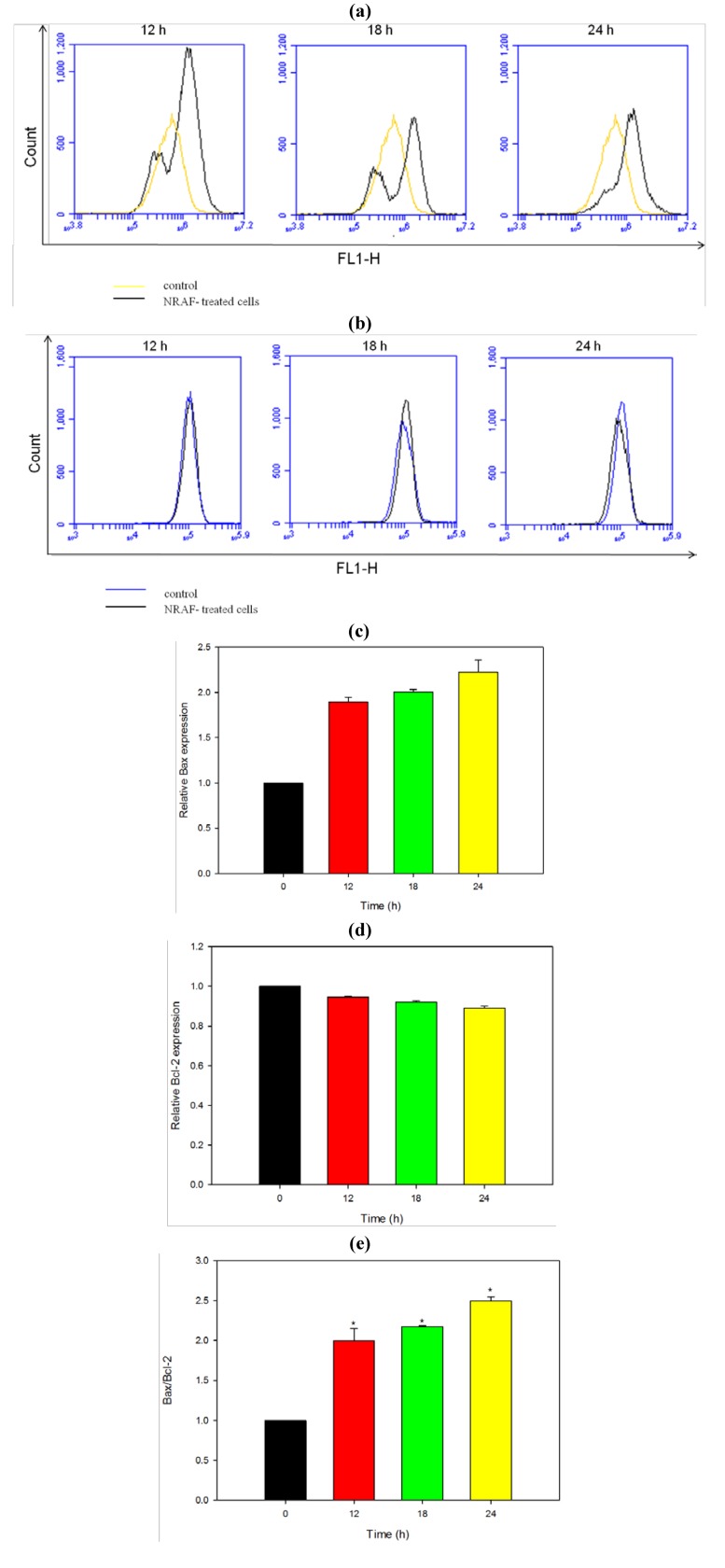
Effect of NRAF on protein expression level of Bax and Bcl-2 in HT-29 cells. Cells were treated with 50 µg/mL of NRAF for different times. (**a**) The histogram showed the expression of Bax in the treated-HT-29 cells at different exposure time. (**b**) The histogram showed the expression of Bcl-2 in the treated-HT-29 cells at different exposure time. (**c**) Bar chart showed the relative Bax protein expression level. (**d**) Bar chart showed the relative Bcl-2 protein expression level. (**e**) Bar chart showed the ratio of Bax/Bcl-2. The data expressed as mean ± S.E. from three individual experiments. Asterisks indicate significantly different value from control (* *p* < 0.001).

Therefore we applied flow cytometric immunofluorescence staining to confirm the expression of Bax and Bcl-2 protein in individual cells. Cells treated with NRAF exhibited a marked increase in fluorescence intensity (Figure 8a) when stained with Bax antibody compared to the control. This result suggests that Bax protein expression increased in a time-dependent manner (Figure 8c) in cells following treatment with 50 µg/mL of NRAF whereas Bcl-2 level remained low throughout the treatment (Figure 8d). The increase of Bax protein expression (Figure 8a) has elevated the Bax/Bcl-2 ratio by 2.5 fold, at 24 h (Figure 8e). 

We postulated that upregulation of Bax protein alone and/or increased Bax/Bcl-2 ratio might be switching on the mitochondria-mediated apoptotic cascade. By upregulating Bax level in HT-29 cells, NRAF may promote the translocation of Bax from the cytosol to the mitochondrial membrane, leading to the release of cytochrome c. This triggers activation of downstream events such as caspase cascade activation that culminates in cellular apoptosis. The cascade is further amplified by the substantial disruption of *Δψm* elicited by NRAF. Collectively, our findings suggested that the apoptosis induced by NRAF in HT-29 cells was mediated via mitochondrial intrinsic pathway. 

### 2.9. NRAF Induced Caspase-3/7 and Caspase 9 Activation

Studies have identified caspases as important mediators of apoptosis induced by various apoptotic stimuli. Caspases are aspartate-directed cysteine proteases that play a key role in the initiation and execution of apoptosis, necrosis and inflammation, failure of which may cause tumor development and several autoimmune diseases [52,53]. Once activated, caspases activate other downstream caspases, leading to the execution stage of apoptosis. We analyzed cell lysates for the activation of caspase-3/7 and caspase-9 after treatment with NRAF for 12, 18 and 24 h.

**Figure 9:**
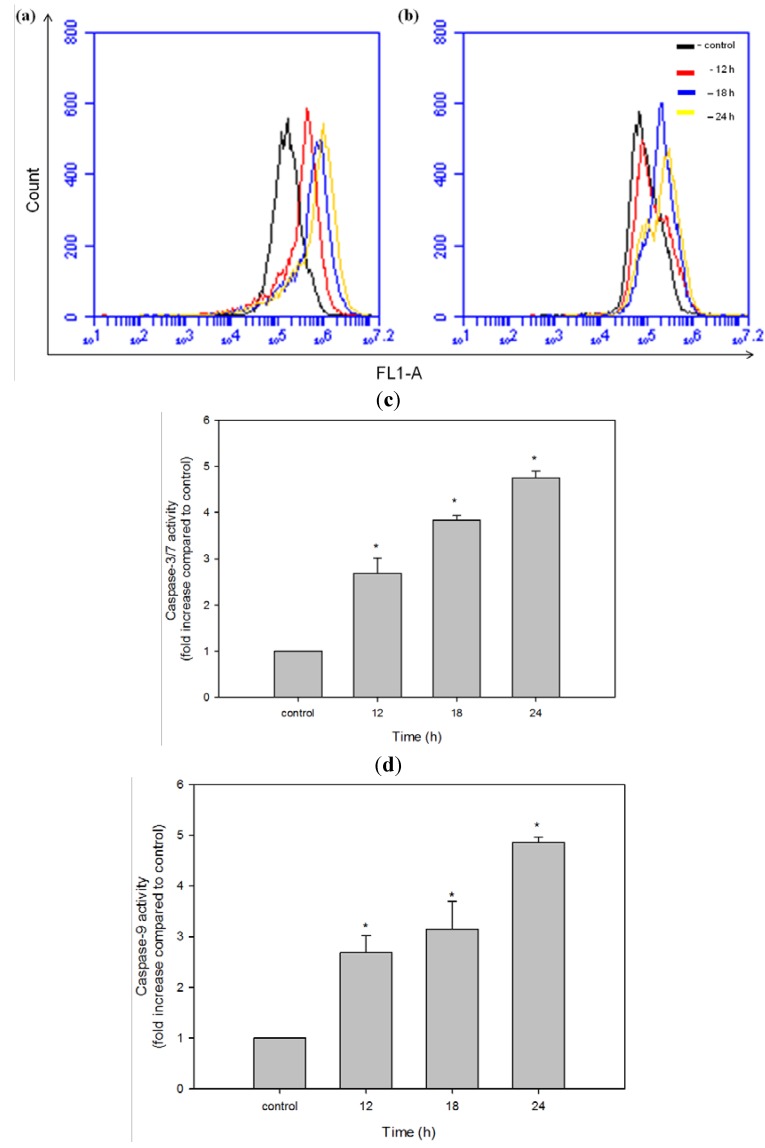
Effect of 50 µg/mL NRAF on caspase activities. (**a**) Indicated the flow cytometric fluorescence patterns of caspase-3/7. (**b**) Indicated the flow cytometric fluorescence patterns of caspase-9. (**c**) Effect of NRAF on the activation of caspase-3/7 in HT-29 cells. (**d**) Effect of NRAF on the activation of caspase-9 in HT-29 cells. Both caspase activities were assessed in time-dependent manner (12, 18 and 24 h) at 50 µg/mL of NRAF. The data expressed as mean ± S.E. from three individual experiments. Asterisks indicate significantly different value from control (* *p* < 0.05).

The ratio of proapoptotic Bax to antiapoptotic Bcl-2 has been reported to be correlated to the initiation of a cascade which leads to the activation of caspases, such as caspase-3/7 [54]. Thus, caspase activation is the key event in apoptotic cell death. Our finding of the NRAF-induced upregulation of Bax protein and the resultant increase in the Bax/Bcl-2 ratio led us investigate the roles of caspase-3/7 and caspase-9 in the NRAF-induced apoptotic pathways. To analyze the apoptotic pathway in NRAF-treated HT-29 cells, we examined the caspase-3/7, caspase-9 and caspase-8 activities using fluorochrome inhibitors, FAM-DEVD-FMK, FAM-LEDH-FMK and FAM-LETD-FMK for caspase-3/7, caspase-9, and caspase-8, respectively. The fluorescence intensity is proportional to the caspase activity. Both caspase-3/7 (Figure 9a) and caspase-9 (Figure 9b) were significantly activated in a time-course study (12, 18 and 24 h incubation) when HT-29 cells were exposed to 50 µg/mL of NRAF as evident in the shift of histogram from left to right indicating an increase in the fluorescence intensity. In contrast, no active form of caspase-8 was observed in these cell lysates (data not shown). In this study, we observed activation of caspase-9 and caspase-3/7, but not caspase-8, after treatment with NRAF. These results suggest that NRAF induces apoptosis in HT-29 cells, accompanied by the trigger of caspase-9 and caspase-3/7 activation. 

## 3. Experimental

### 3.1. Plant Material

The *N. ramboutan-ake (Syn. Nephelium mutabile)* used in this study was identified by Dr Yong Kien Tai, curator of herbarium of the University of Malaya. The voucher specimen of *N. ramboutan-ake* was deposited and numbered No.KLU 47703 in the herbarium of the University of Malaya.

### 3.2. Preparation of Crude *N. ramboutan-ake* Extract and Fractions

Powdered samples (1.2 kg) were soaked with 95% ethyl alcohol (2.0 L, three times) at room temperature for 3 days. The extract obtained was filtered from the residue through Whatman No.1 filter paper. The filtrate was concentrated using a rotary evaporator (Büchi) under reduced pressure at 40 °C. The dark red crude extract (35.81 g) which was in powder form was obtained and stored in specimen vials. The ethanol extract was partitioned with hexane (250 mL, room temperature). The hexane insoluble fraction was further partitioned with ethyl acetate and water in 1:1 ratio (500 mL: 500 mL, room temperature) to yield an ethyl acetate-soluble fraction. The ethyl acetate-soluble fraction was evaporated under reduced pressure at 45 °C, while the aqueous filtrate was lyophilized to yield the aqueous fraction. The *N. ramboutan-ake *rind ethanol extract (NREE), *N. ramboutan-ake *rind ethyl acetate fraction (NREAF) and *N. ramboutan-ake *rind aqueous fraction (NRAF) were dissolved in DMSO prior to each assay. The final concentration of DMSO in all the experiments did not exceed 0.5% v/v. All samples were filter sterilized with 0.22 µm filters before use.

### 3.3. Cell Culture

In the present study, the HT-29, HCT-116, Ca Ski and MDA-MB-231 were obtained from the American Type Culture Collection (ATCC, Manassas, VA, USA). All cells were maintained in RPMI 1640 medium (Sigma, St. Louis, MO, USA). All medium were supplemented with 10% v/v heat-inactivated fetal bovine serum (PAA Lab, Pasching, Austria), 100 µg/mL penicillin (PAA Lab) and 50 µg/mL amphotericin B (PAA Lab). The media was filter sterilized using a 0.22 µm filter membrane (Minisart, Sartorius Stedim, Goettingen, Germany). The cells were cultured in 5% CO_2_ incubator at 37 °C in a humidified atmosphere. The culture was sub cultured every 2 or 3 days and routinely checked under an inverted microscope for any contamination. Cells in the exponential growth phase were used for all experiments. The cells were harvested from culture flasks by Accutase (Innovative Cell Technologies, San Diego, CA, USA) and the viable cell count was determined using tryphan blue exclusion assay with haemocytometer. 

### 3.4. *In Vitro* MTT Cytotoxicity Assay

The cell viability was investigated using the MTT assay. Viable cells were seeded into 96-well plates and were allowed to adhere overnight prior to treatment with various concentrations (3.125–200 µg/mL) of *N. ramboutan-ake *rind extract and fractions. For the untreated cells (control), vehicle dimethyl sulfoxide (DMSO) was added instead of the sample. After 72 h incubation, MTT (20 µL, 5 mg/mL, Sigma) was added to each well and the plates were incubated for another 4 h at 37 °C. Following incubation, the culture medium was removed by gentle aspiration and replaced with DMSO (150 µL), to dissolve the formazan crystals. The amount of formazan product was measured at 570 nm and 650 nm as a background using a microplate reader (Asys UVM340, Eugendorf, Austria). The percentage of cell viability = (absorbance of treated cells/absorbance of untreated cells) × 100% 

### 3.5. Preliminary Phytochemical Screening

The aqueous fraction of *N. ramboutan-ake* was subjected to preliminary qualitative phytochemical screening according to the Sofowora, Trease and Bibi methods [55,56,57]. This analysis was to determine the presence of phytoconstituents such as flavonoids, tannins, saponins, alkaloids and sterols.

#### 3.5.1. Test for Alkaloids

The extract (0.5 g) was added to 1% aqueous hydrochloric acid (5 mL) on a steam bath and filtered. A few drops of Dragendorff’s reagent were added into 1 mL of the filtrate. Turbidity or precipitation with this reagent was taken as evidence for the presence of alkaloids. 

#### 3.5.2. Sakowski Test for Sterols

The plant extract (0.5 g) was dissolved in chloroform (1 mL); concentrated sulphuric acid (1 mL) was added carefully along the sides of the test tube. Production of a red colour indicates the presence of steroids.

#### 3.5.3. Frothing Test for Saponins

Plant extract (0.5 g) was dissolved in boiling water (1 mL) followed by cooling (room temperature) and shaking. Appearance of froth indicates the presence of saponins.

#### 3.5.4. Test for Tannins

Plant extract (0.5 g) was boiled in distilled water (20 mL) in a test tube and then filtered. Two to three drops of FeCl_3_ (0.1%) was added to filtrate. Appearance of brownish green or blue black coloration showed the presence of tannins.

#### 3.5.5. Test for Flavonoids

Prepared extract (0.5 g) was shaken with petroleum ether (5 mL) to remove the fatty materials. The defatted residue was dissolved in 80% of ethanol (20 mL) and filtered. The filtrate (3 mL) was mixed with 1% KOH (4 mL). A dark yellow color was observed indicating the presence of flavonoids

### 3.6. Nuclear Morphology Detection Using Hoechst 33342/PI

HT-29 cells (1 × 10^6^ cells/mL) were seeded in 60 mm^2^ culture dish and treated with NRAF or vehicle DMSO (control). After indicated periods, the cells were harvested and washed with PBS. The cells were stained with Hoechst 33342 and PI solution at 37 °C in dark for 30 min. Then, the cells were placed observed under inverted fluorescence microscope (Leica DM16000B, Wetzlar, Germany).

### 3.7. Terminal Deoxynucleotidyl Transferased UTP Nick End Labeling (TUNEL) Assay

For detection of DNA breakage, a TUNEL assay was performed following the protocol provided by the manufacturer (Sigma). In brief, HT-29 cells (1 × 10^6^ cells/mL) were seeded in 60 mm^2^ culture dish and treated with NRAF or vehicle DMSO (control). NRAF-treated cells were harvested, washed with PBS and fixed with 1% (w/v) paraformaldehyde in PBS on ice for 15 min. After fixation, the cells were washed and then incubated in DNA labeling solution [containing terminal deoxynucleotidyl transferase enzyme, bromodeoxyuridine (BrdU), and TdT reaction buffer] for 60 min at 37 °C. The cells were then rinsed and incubated with FITC-conjugated anti-BrdU antibody for 30 min at room temperature in the dark. Subsequently, the propidium iodide/RNase A solution was added to the cells and further incubated for another 30 min in the dark. The cells were then analysed by using Accuri C6 flow cytometry and the fluorescence intensity in X-axis and Y-axis were detected in FL1-A and FL2-A channel respectively.

### 3.8. Annexin V/PI Staining for the Assessment of Phosphatidylserine Externalization

The early and late apoptosis induced by NRAF was further investigated using Annexin V/PI staining. Thus, HT-29 cells (1 × 10^6^ cells/mL) were seeded in 60 mm^2^ culture dish and treated with NRAF or vehicle DMSO (control). After HT-29 cells (1 × 10^6^ cells/mL) were treated with NRAF, both adherent and suspension cells were harvested, washed twice with PBS, re-suspended in annexin V binding buffer (BD) and stained at room temperature in the dark for 30 min with annexin V-FITC (BD) and PI (Sigma) as described in the manufacturer’s protocol. After treatment with various concentration of NRAF ranging 25 µg/mL to 200 µg/mL, the HT-29 cells were then analyzed by flow cytometry using quadrant statistics for apoptotic and necrotic cell populations. The fluorescence intensity in X-axis and Y-axis were detected in FL1-A and FL2-A channel respectively. Annexin V was used to detect both the early and late stages of apoptosis while PI was used to detect late apoptosis and necrosis. The discrimination between viable (both annexin V and PI negative), late apoptosis (both annexin V and PI positive), and necrotic (annexin V negative and PI positive) cells was achieved by quantitatively estimating the relative amounts of the annexin V/PI-stained cells in the cell population.

### 3.9. Measurement of Mitochondrial Membrane Potential (Δψm)

The change in mitochondrial membrane potential (*Δψm*) was assessed by using the cell-permeable, mitochondrial-specific fluorescent probe JC-1 dye. As usual, HT-29 cells (1 × 10^6^ cells/mL) were seeded in 60 mm^2^ culture dish and treated with NRAF. For the untreated cells (control), vehicle dimethyl sulfoxide (DMSO) was added instead of the sample. After treatment with NRAF, the cells were harvested, washed and stained with medium containing JC-1. The cells were incubated at 37 °C in the incubator for 15 min. Subsequently, the cells were washed again and resuspended in the medium. Finally the cells were subjected to flow cytometry analysis by detecting the green and red fluorescence signals. JC-1 aggregates in the mitochondria of healthy cells produced red fluorescence as observed in FL2-A channel while in the cells with altered mitochondrial membrane potentials, the JC-1 dye maintained as monomeric form in the cytoplasm where it fluoresced green and detected in FL1-A channel.

### 3.10. Determination of Intracellular Total Glutathione (GSH) Content

Intracellular GSH level was determined after treatment with various concentrations ranging from 50 µg/mL to 200 µg/mL of NRAF and vehicle DMSO (control). The treated cells were harvested, centrifuged, washed with ice-cold PBS and re-suspended in 500 µL of 5% 5-sulfosalicyclic acid before incubating on ice for 15 min with intermittent vortexing. The cell suspension was centrifuged at 10,000 × g to collect the supernatant. The supernatant (10 µL) was then subjected to glutathione assay in 96-well plate format in a 150 µL of working solution. The final concentrations of the reaction mixture were 95 mM potassium phosphate buffer (pH 7.0), 0.95 mM EDTA, 228 µL of glutathione reductase enzyme (6 unit/mL) and 228 µL of 5,5'-dithiobis-2-nitrobenzoic acid (DTNB 1.5 mg/mL) were then added and left for 5 min prior to addition of 50 µL of NADPH solution (0.16 mg/mL). Absorbance was measured at 1 min intervals for 10 min at 412 nm with an Oasys UVM340 microplate reader and compared with a glutathione standard curve. The results were expressed as pmoles of glutathione per milligram of protein (pmole GSH/mg protein).

### 3.11. Measurement of Intracellular Reactive Oxygen Species (ROS)

The fluorescent probe 2'-7'-dichlorofluorescein diacetate (DCFH-DA) used to monitor intracellular accumulation of ROS. HT-29 cells (1 × 10^6^ cells/mL) were seeded in 60 mm^2^ culture dish and treated with NRAF or vehicle DMSO (control) while *tert*-butyl hydroperoxide (TBHP) was served as positive control. After 4 h treatment with various concentrations (50 µg/mL to 200 µg/mL) of NRAF, the cells were washed and incubated with medium containing 50 µM DCFH-DA for 1 h. Cells were then harvested and washed again with PBS. The cell suspension was re-suspended in PBS and the fluorescence intensity was measured by flow cytometry and detected in FL1-A channel. 

### 3.12. Determination of Bax and Bcl-2 Protein Expression Level

The protein expression level of Bax and Bcl-2 was assessed by immunofluorescence staining using flow cytometry. This method was based on Roussi *et al*. [58] with some modifications. After HT-29 cells (1 × 10^6^ cells/mL) were treated with NRAF or DMSO (control), both adherent and suspension cells were harvested, washed twice with PBS and then fixed and permeabilized using the Cytofix/Cytoperm kit (BD Biosciences, San Jose, CA, USA). NRAF-treated cells (1 *× *10^6^ cells) were resuspended in fixation/permeabilization solution (500 µL) and incubated for 20 min at 4 °C. The cells were washed twice with Perm/Wash buffer and incubated for 15 min in this buffer (1 mL). To detect Bax or Bcl-2, the fixed and permeabilized cells were incubated with Perm/Wash buffer (100 µL) containing the antibodies. For Bcl-2 protein, the cells were stained directly for 30 min with FITC-conjugated mouse anti-human Bcl-2 monoclonal antibody or IgG1 isotype control (10 μL, BD Biosciences) at 4 °C. For indirect Bax staining, the cells were incubated for 30 min with either rabbit anti-human Bax polyclonal antibody or IgG1 isotype control (BD Biosciences) at 4 °C. After washing, the cells were further incubated for 30 min with FITC-conjugated goat anti-rabbit F(ab')2 polyclonal secondary antibody (Abcam) at 4 °C. The cells were then washed with Perm/Wash buffer and analyzed using C6 Accuri flow cytometer and detected in FL1-H channel.

### 3.13. Measurement of Caspase-3/7 and Caspase-9 Activities

HT-29 cells (1 × 10^6^ cells/mL) were seeded in 60 mm^2^ culture dish and treated with NRAF or vehicle DMSO (control). After treatment, cells were harvested and cell suspensions were stained with 30× FLICA solution (caspase 3/7-FAM-DEVD-FMK; caspase 9-FAM-LEHD-FMK; caspase 8-FAM-LETD-FMK) for 1 h at 37 °C under 5% CO_2_ in darkness. Cells were then washed twice with wash buffer followed by resuspending of cell pellet in wash buffer. FAM-DEVD-FMK FLICA, FAM-LEHD-FMK and FAM-LETD-FMK will bind to caspase-3/7, caspase-9 and caspase-8, respectively that are present in the cells and appear as green fluorescence. Increase in fluorescence intensity indicating caspase-3/7, caspase-9 and caspase-8 activities were detected by flow cytometry and detected in FL1-A channel.

### 3.14. Statistical Analysis

In all the experiments, data were expressed as means ± standard error. A significant difference from the respective control for each experiment was assessed using Student’s *t*-test, with *p* values <0.05 being regarded as statistically significant. 

## 4. Conclusions

The results of the present study suggest that NRAF may be useful for integrative and complementary medicine by promoting apoptotic cell death in HT-29 human colorectal cancer cells via induction of ROS production, GSH depletion, mitochondrial dysfunction, proapoptotic Bax protein modulation and caspase activation.
